# Direct generation of massive vector-mode entanglement from a polarization-insensitive optical amplifier

**DOI:** 10.1126/sciadv.aec0001

**Published:** 2026-04-17

**Authors:** Xutong Wang, Yao Wei, Kai Zhang, Xiaozhou Pan, Jietai Jing

**Affiliations:** ^1^ State Key Laboratory of Precision Spectroscopy, Joint Institute of Advanced Science and Technology, School of Physics and Electronic Science, East China Normal University, Shanghai 200062, China.; ^2^ CAS Center for Excellence in Ultra-intense Laser Science, Shanghai 201800, China.; ^3^ Collaborative Innovation Center of Extreme Optics, Shanxi University, Taiyuan, Shanxi 030006, China.; ^4^ Institute of Nonlinear Physics and Department of Physics, Zhejiang Normal University, Jinhua, 321004 Zhejiang, China.

## Abstract

Vectorially structured modes, with spatially inhomogeneous polarization, have attracted widespread attention due to their diverse applications across various physical processes. Entangling these modes holds great potential for advancing quantum optical technologies. In this work, we present a polarization-insensitive amplifier based on the four-wave mixing process, capable of directly generating large numbers of entangled vector vortex (VV) modes. When seeded by vacuum fields, the amplifier simultaneously produces 16 sets of two-mode squeezed vacuum states or continuous-variable entangled states, each containing two VV modes. This work opens avenues for developing quantum information processing that fully harnesses the vectorial nature of optical fields.

## Teaser

A polarization-insensitive amplifier is used to directly generate quantum entanglement between vectorially structured modes.

## INTRODUCTION

Optical systems, owing to their advantages as quantum platforms with high coherence, robustness under ambient conditions, and suitability for long-distance information transmission, have become a natural choice for numerous quantum applications. Tailoring the structure of optical fields across multiple degrees of freedom, such as polarization, amplitude, and phase, and increasing their complexity have driven substantial advancements in both fundamental research and applied technology in modern optics (*1*–*3*). For instance, in the classical domain, this approach enables enhanced microscopy, metrology, and communication capabilities, while in the quantum domain, it provides a platform for exploring the foundations of quantum mechanics and facilitates quantum information processing with improved information capacity and noise tolerance.

Optical polarization, which defines the oscillation direction of electric field, is typically homogeneous across the spatial profile of an optical beam, a configuration known as a scalar optical field. However, polarization can also be spatially inhomogeneous, giving rise to vector optical fields with distinctive polarization patterns (*4*–*10*). In general, vector optical fields are characterized by inhomogeneous polarization. Among the various types of vector optical fields, vector vortex (VV) modes, often generated by superposing two distinct scalar orbital angular momentum (OAM) modes with orthogonal circular polarizations, represent a special class of vector optical fields. In particular, cylindrically symmetric VV modes, such as the widely studied radially and azimuthally polarized VV modes, form a prominent subclass of VV modes and can exhibit central polarization singularities (*11*).

VV modes have found widespread use in numerous applications, including imaging (*12*), metrology (*13*, *14*), and communications (*15*–*17*). The entanglement of these modes has drawn considerable interest due to its potential in quantum technological applications (*18*–*28*). However, generating VV-mode entanglement remains challenging because of the complex, spatially inhomogeneous polarization patterns of these modes. This difficulty arises from the fact that previously used nonlinear optical processes for generating quantum entanglement, such as parametric down-conversion or four-wave mixing (FWM), are typically polarization dependent due to the phase-matching conditions, which constrains the polarization patterns of the generated optical fields. Entangled VV modes have been demonstrated by converting polarization-entangled photon pairs into entangled VV photon pairs through additional optical components (*21*, *23*, *27*). However, these approaches require intricate optical setups beyond the nonlinear process itself, which introduces additional optical losses or demands long-term interferometric stability. For direct generation of VV-mode entanglement from a nonlinear optical process, the process must operate independently of the polarization patterns, meaning that it must be polarization independent.

In this work, we propose a polarization-insensitive optical amplifier scheme that enables the direct generation of massive vector-mode entanglement from a single nonlinear process. By leveraging the intrinsic conservation of spin angular momentum (SAM) and OAM in the FWM process in a hot ^85^Rb atomic vapor, we design a polarization-insensitive and spatially multimode parametric amplifier capable of mixing and amplifying two vectorially structured optical fields while preserving their complex polarization patterns. When seeded by vacuum states, the amplifier produces two-mode squeezed vacuums, or continuous-variable (CV) entanglement, of vector optical fields. In our experiment, we confirm the direct generation of 16 pairs of entangled VV modes from this amplifier.

## RESULTS

### Principles of the polarization-insensitive amplifier

**Fig. 1.**
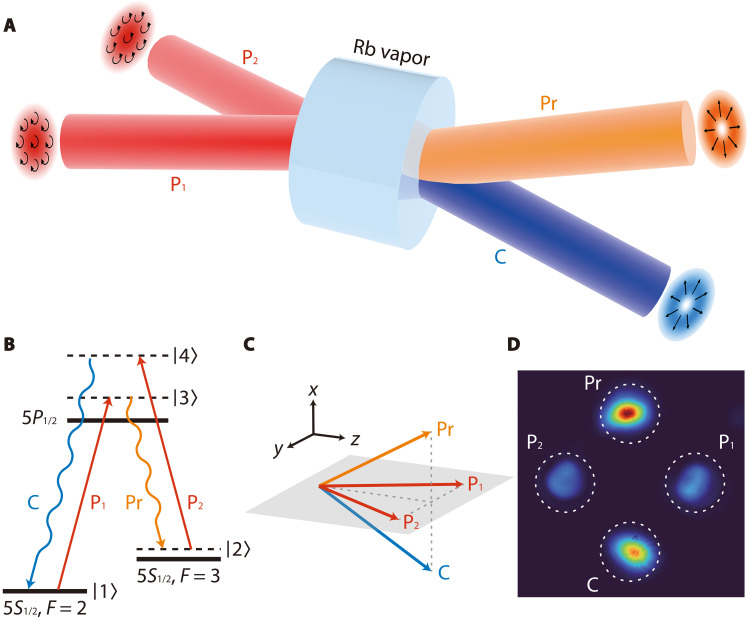
Schematic illustration of the FWM process for constructing a polarization-insensitive amplifier. (**A**) Two pump beams (P_1_ and P_2_) intersect at the center of an Rb atomic vapor cell, leading to the generation of probe (Pr) and conjugate (C) beams through the FWM process. (**B**) Double-Λ energy level configuration in the *D*1 line of ^85^Rb. (**C**) Spatial geometry of the four interacting beams (not to scale). (**D**) Far-field intensity distributions after the FWM process. The two pump beams are strongly attenuated after the vapor cell to prevent overexposure of the CCD camera.

The FWM process for constructing a polarization-insensitive amplifier is schematically shown in Fig. 1A, where pump photons are annihilated, leading to the creation of correlated probe and conjugate photons in a ^85^Rb atomic vapor cell. Following a double-Λ energy level configuration in the *D*1 line of ^85^Rb (Fig. 1B), the pump beam P_1_ drives an off-resonant transition from the *F* = 2 (5*S*_1/2_) ground state |1⟩ to the virtual state |3⟩ which is 0.95 GHz blue-shifted from the excited state 5*P*_1/2_. This results in the spontaneous emission of a probe photon through a transition from virtual state |3⟩ to another virtual state |2⟩ which is 10 MHz blue-shifted from the *F* = 3 (5*S*_1/2_) ground state. The pump beam P_2_, with the same frequency as P_1_, then drives a second off-resonant transition |2⟩ → |4⟩, leading to the subsequent spontaneous emission of a conjugate photon and the return of the atom to the ground state |1⟩. In this parametric process, total angular momentum is conserved. In addition, the homogeneous and isotropic atomic medium, along with paraxial optical beams that are not tightly focused, ensures the independent conservation of both SAM and OAM. Specifically, when the two pump beams are fundamental Gaussian modes with orthogonal circular polarizations, the interaction Hamiltonian of this FWM process, under the undepleted pump approximation, can be written as

$$\hat{H}=\sum_{\ell }i\hbar \gamma_{\ell }\left({\hat{a}}_{R,\ell }^{†}{\hat{b}}_{L,-\ell }^{†}+{\hat{a}}_{L,\ell }^{†}{\hat{b}}_{R,-\ell }^{†}\right)+H.C$$ (1)

where ${\hat{a}}_{R,\ell }^{†}$ (${\hat{a}}_{L,\ell }^{†}$) and ${\hat{b}}_{L,-\ell }^{†}$ (${\hat{b}}_{R,-\ell }^{†}$) are the creation operators of probe and conjugate modes, respectively, with the two subscripts denoting their SAM (R, right-circular polarization; L, left-circular polarization) and OAM (ℓ, topological charge), $\gamma_{\ell }$ represents the corresponding interaction strength, and H.C. is the Hermitian conjugate. The input-output relations for probe and conjugate fields are then given by

$$\begin{array}{c} {\hat{a}}_{R,\ell }^{†}=\sqrt{G_{\ell }}{\hat{c}}_{R,\ell }^{†}+\sqrt{G_{\ell }-1}{\hat{d}}_{L,-\ell },\\ {\hat{b}}_{L,-\ell }^{†}=\sqrt{G_{\ell }-1}{\hat{c}}_{R,\ell }+\sqrt{G_{\ell }}{\hat{d}}_{L,-\ell }^{†} \end{array}$$ (2)

and

$$\begin{array}{c} {\hat{a}}_{L,\ell }^{†}=\sqrt{G_{\ell }}{\hat{c}}_{L,\ell }^{†}+\sqrt{G_{\ell }-1}{\hat{d}}_{R,-\ell },\\ {\hat{b}}_{R,-\ell }^{†}=\sqrt{G_{\ell }-1}{\hat{c}}_{L,\ell }+\sqrt{G_{\ell }}{\hat{d}}_{R,-\ell }^{†} \end{array}$$ (3)

where ${\hat{c}}_{R,\ell }^{†}$ (${\hat{c}}_{L,\ell }^{†}$) and ${\hat{d}}_{L,-\ell }^{†}$ (${\hat{d}}_{R,-\ell }^{†}$) represent the creation operators for the input probe and conjugate modes, respectively, $G_{\ell }={\text{cosh}}^{2}\left(\gamma_{\ell }\tau \right)$ corresponds to the intensity gain, and τ is the interaction time. Therefore, the above FWM process can function as an optical amplifier that operates on optical modes with complex SAM-OAM configurations, following the superposition of Eqs. 2 and 3. By contrast, when both pump beams carry the same circular polarization, the FWM process obeys a definite polarization selection rule and is no longer polarization insensitive (see section S1 of the Supplementary Materials).

When the input probe and conjugate fields are both in vacuum states, the amplifier based on the FWM process mixes and amplifies these two vacuum fields, generating vacuum twin beams that contain massive two-mode squeezed vacuum states (*29*, *30*). Here, the amplitude quadrature $\hat{x}=\hat{o}+{\hat{o}}^{†}$ and phase quadrature $\hat{p}=i\left({\hat{o}}^{†}-\hat{o}\right)$, both of which can be defined as continuous superpositions of eigenstates (*31*), are used to quantum-mechanically describe each optical mode, where ${\hat{o}}^{†}$ is the creation operator of corresponding mode. For each two-mode squeezed vacuum state, there is correlation between the amplitude quadratures and anticorrelation between the phase quadratures of the two modes, a phenomenon known as CV entanglement. Such CV entanglement can be tested by constructing the covariance matrix (CM) based on the quadratures and calculating the smallest symplectic eigenvalue of the partially transposed (PT) CM. According to the positivity under partial transposition (PPT) criterion (*32*), a smaller symplectic eigenvalue corresponds to a stronger degree of CV entanglement, with a value less than one confirming the existence of CV entanglement.

In our experiment, as shown in Fig. 1C, the two pump beams, each with a power of 300 mW, form the *yz* plane and intersect at an angle of 17.4 mrad at the center of the hot ^85^Rb atomic vapor cell which is heated to 118°C. The probe and conjugate beams symmetrically intersect the pump beams, with an angle of about 8.5 mrad between them and the *yz* plane. To better illustrate the spatial geometry of the four interacting beams, we present their experimental far-field intensity distributions, as shown in Fig. 1D, which were captured by a charge-coupled device (CCD) camera after seeding a bright beam along the direction of the probe beam to amplify it and generate a bright conjugate beam through FWM process. This geometry ensures that the desired two-pump FWM process achieves a relatively high intensity gain while strongly suppressing unwanted single-pump FWM interactions (*29*) between the probe (or conjugate) beam and a single pump beam due to phase-matching constraints (*33*), which could otherwise introduce unwanted photons into the probe (or conjugate) field (see section S2 of the Supplementary Materials).

### Experimental verification of the polarization-insensitive property

**Fig. 2.**
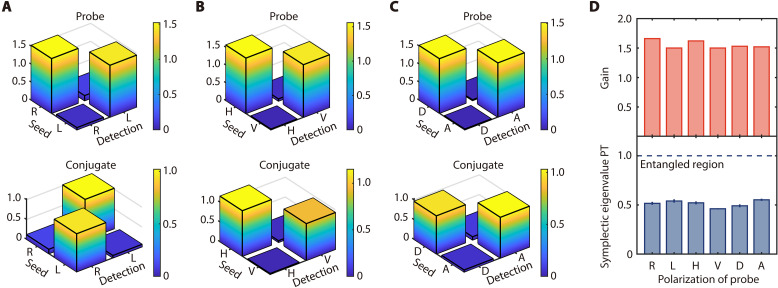
Demonstration of the polarization-insensitive property of the amplifier. (**A** to **C**) Polarization analysis of the amplified probe beam (top) and the generated conjugate beam (bottom) from the amplifier when the seed probe beam is right-circularly (R), left-circularly (L), horizontally (H), vertically (V), diagonally (D), and antidiagonally (A) polarized. (**D**) Intensity gain of the amplifier (top) and the smallest symplectic eigenvalue of PT CM (bottom) for different polarizations. The uncertainties represent one SD derived from multiple repeated measurements.

To demonstrate the polarization-insensitive property of this amplifier, we seed a bright fundamental Gaussian beam with varying polarizations into the amplifier along the direction of the probe beam and analyze the polarization of the output probe and conjugate beams. When the seed probe beam is right- or left-circularly polarized, the intensity of the different polarization components, normalized to the intensity of the seed probe beam, for the output probe and conjugate beams is shown in Fig. 2A. The amplified probe beam exhibits a high degree of circular polarization, maintaining the same polarization as the seed probe beam. In addition, the generated conjugate beam has the opposite circular polarization to the probe beam, indicating the conservation of SAM in the FWM process. Furthermore, we repeat the same measurements when the seed probe is linearly polarized (i.e., horizontal, vertical, diagonal, and antidiagonal polarizations), which can be expressed as equal superpositions of right- and left-circular polarization, as shown in Fig. 2 (B and C). For all these cases, the seed probe beam is amplified and retains its original polarization, while the generated conjugate beam matches the seed probe’s polarization. On the basis of the results in Fig. 2 (A to C), we plot the intensity gain of the amplifier, defined as the ratio of the amplified probe beam intensity to the seed probe beam intensity, for different seed probe polarizations, as shown in the top of Fig. 2D. Across all these polarizations, the intensity gain remains nearly constant, confirming the polarization-insensitive operation of the amplifier.

As mentioned above, when the input probe and conjugate fields are both in vacuum states, this polarization-insensitive amplifier can be used to generate two-mode squeezed vacuums, which manifests as entanglement between the quadratures of two polarization modes simultaneously generated in the FWM process. To experimentally measure the amplitude and phase quadratures and verify CV entanglement, we use two sets of balanced homodyne detection (BHD), one for the probe and the other for the conjugate. In each BHD, the output optical fields are projected onto a specific polarization mode determined by the polarization of the local oscillator. The corresponding symplectic eigenvalues are then obtained from the experimentally measured quadratures, as shown in the bottom of Fig. 2D. The results show that the symplectic eigenvalue remains nearly unchanged across different polarization modes. Therefore, the polarization-insensitive property of this amplifier is further verified from the perspective of CV quantum systems, demonstrating that the amplifier can generate CV entanglement with different polarization modes while maintaining an almost constant degree of entanglement.

### Generation of entangled VV modes

Furthermore, by leveraging the polarization-insensitive property, in conjunction with the spatially multimode property observed in the same energy level configuration (*29*, *30*), we explore the operation of this amplifier on optical modes with complex SAM-OAM configurations. Specifically, we focus on a class of cylindrically symmetric VV modes expressed in the Laguerre-Gaussian mode basis as

$$E_{\ell ,\pm }\left(r,\phi ,z\right)={\text{LG}}_{\ell }\left(r,\phi ,z\right)e_{R}\pm {\text{LG}}_{-\ell }\left(r,\phi ,z\right)e_{L}$$ (4)

where $e_{R}$ and $e_{L}$ represent the circular polarization bases, and ${\text{LG}}_{\ell }\left(r,\phi ,z\right)$ denotes the complex amplitude of a Laguerre-Gaussian mode with topological charge ℓ and a radial index of 0. The mode orders (ℓ,±) define a specific VV mode, and modes with different orders are orthogonal to each other. Correspondingly, we introduce the creation operators of the VV modes defined in Eq. 4 as a unitary transformation of the SAM-OAM basis: ${\hat{f}}_{\ell ,\pm }^{†}=\left({\hat{a}}_{R,\ell }^{†}\pm {\hat{a}}_{L,-\ell }^{†}\right)/\sqrt{2}$, ${\hat{g}}_{\ell ,\pm }^{†}=\left({\hat{b}}_{L,-\ell }^{†}\pm {\hat{b}}_{R,\ell }^{†}\right)/\sqrt{2}$, ${\hat{h}}_{\ell ,\pm }^{†}=\left({\hat{c}}_{R,\ell }^{†}\pm {\hat{c}}_{L,-\ell }^{†}\right)/\sqrt{2}$, and ${\hat{i}}_{\ell ,\pm }^{†}=$ $\left({\hat{d}}_{L,-\ell }^{†}\pm {\hat{d}}_{R,\ell }^{†}\right)/\sqrt{2}$. Assuming equal intensity gain for opposite OAM orders ($G_{\ell }=G_{-\ell }$), which is valid since these modes have identical beam size and therefore similar overlap with the pump beams (*30*), substituting these definitions into the input-output relations in the SAM-OAM basis (Eqs. 2 and 3) yields the input-output relations in the VV mode basis

$$\begin{array}{c} {\hat{f}}_{\ell ,\pm }^{†}=\sqrt{G_{\ell }}{\hat{h}}_{\ell ,\pm }^{†}+\sqrt{G_{\ell }-1}{\hat{i}}_{\ell ,\pm },\\ {\hat{g}}_{\ell ,\pm }^{†}=\sqrt{G_{\ell }-1}{\hat{h}}_{\ell ,\pm }+\sqrt{G_{\ell }}{\hat{i}}_{\ell ,\pm }^{†} \end{array}$$ (5)

showing that the polarization-insensitive amplifier thus mixes and amplifies VV mode pairs sharing the same mode orders (ℓ,±). Such input-output relations in Eq. 5 correspond to Bogoliubov transformations associated with two-mode squeezing interaction, which can generate CV entangled states (see section S3 of the Supplementary Materials for a detailed derivation) (*31*).

**Fig. 3.**
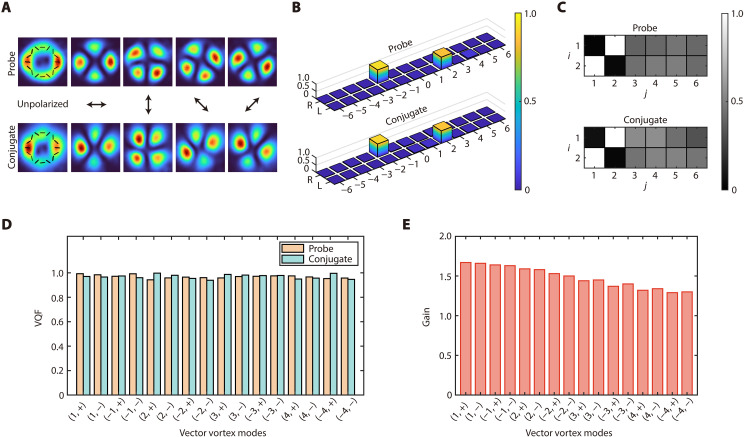
Amplification of vector modes. (**A**) Mode structure analysis of the amplified probe beam (top) and the generated conjugate beam (bottom) from the amplifier when seeding the VV mode (−2,+) into the amplifier along the direction of the probe beam. (**B**) OAM modal analysis of the VV modes in (A) under right- and left-circular polarization. (**C**) Measured normalized projection intensities $I_{\mathrm{ij}}$ used to evaluate the VQF of the VV modes in (A). See Table 1 in Materials and Methods for the definitions of indices i and j. (**D**) VQF for the generated VV modes with different mode orders. (**E**) Intensity gain of the amplifier for the VV modes with different mode orders.

To demonstrate the amplification of these VV modes, we seed a bright beam with controllable mode orders into the amplifier along the direction of the probe beam and analyze the mode structure of the output probe and conjugate beams (see Materials and Methods for details about the experimental setup). For instance, when the amplifier is seeded by the VV mode (−2, +), Fig. 3A presents the experimentally obtained intensity distributions and polarization patterns of the output probe and conjugate beams (leftmost panel), as well as their transmitted intensity distributions under various linear polarization projections (four rightmost panels). It is clearly observed that under linear polarization projection, the doughnut-shaped output probe and conjugate beams split into four (i.e., 2∣ℓ∣) lobes that rotate with the linear polarization, which is a consequence of the spatially variant polarization of the VV mode. These spatially variant polarization patterns indicate that both the output probe and conjugate beams are VV modes with the same mode orders as the seed beam. In addition, to quantify the OAM component and modal purity, we analyze the output beams under circular polarization projection using OAM modal analysis (*34*). The resulting OAM spectrum directly yields the fractional contribution of OAM components and thus the OAM modal purity. Figure 3B shows the OAM modal analysis results for the VV modes shown in Fig. 3A. These experimental results confirm that, for both the output probe and conjugate beams, the right- and left-circularly polarized components exhibit dominant OAM components of ℓ=−2 and ℓ=2, respectively, consistent with the expected SAM-OAM configuration of the VV mode (−2,+). The corresponding OAM modal purity is 0.93 (right-circular) and 0.85 (left-circular) for the probe beam and 0.90 (right-circular) and 0.86 (left-circular) for the conjugate beam. The OAM modal analysis results for other VV modes are provided in figs. S3 and S4 of the Supplementary Materials. Furthermore, we quantify the degree of vectorness of the output probe and conjugate beams by measuring the vector quality factor (VQF) (*6*, *35*), a metric that ranges from 0 for purely scalar fields to 1 for ideally vectorial fields. The value of each VQF is calculated from 12 normalized intensities corresponding to six spatial-mode projections performed under two circular polarization basis states (see Materials and Methods for details about the measurements). For the VV modes shown in Fig. 3A, the measured normalized projection intensities for the probe and conjugate beams are shown in Fig. 3C, from which VQF values of 0.96 and 0.95 are obtained, respectively. The measured normalized projection intensities used to evaluate the VQF values for other VV modes are provided in figs. S5 and S6 of the Supplementary Materials. As summarized in Fig. 3D, the measured VQF values for all studied VV modes exceed 0.93, quantitatively confirming the vectorial nature of the optical fields generated from the polarization-insensitive amplifier. Moreover, we vary the mode orders of the seed probe beam and record the amplifier’s intensity gain for different VV modes, as shown in Fig. 3E. It is observed that the intensity gain decreases with increasing ∣ℓ∣, which is attributed to the fact that VV mode with a higher value of ∣ℓ∣ has a larger beam size, resulting in reduced overlap with the two pump beams and, consequently, weaker interaction strength. In future work, using two pump beams with flat-top profiles could potentially enable mode order–insensitive amplification of VV modes.

**Fig. 4.**
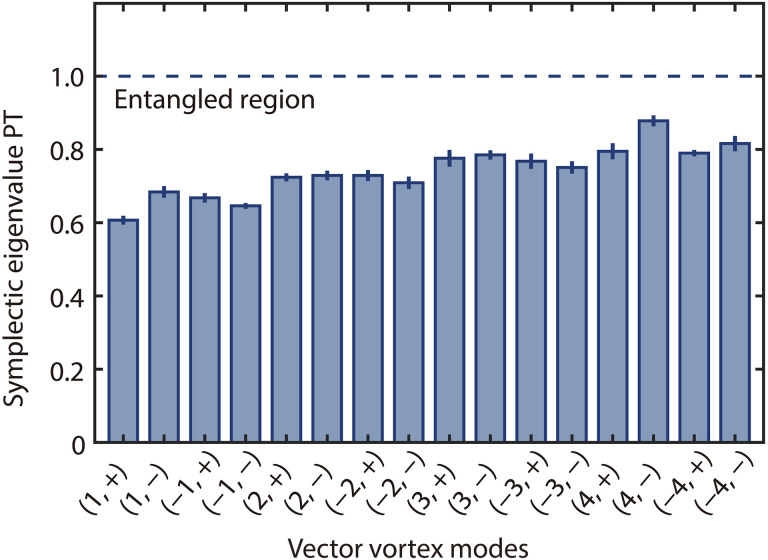
Experimental results for verifying the entanglement between VV modes. The smallest symplectic eigenvalue of PT CM for the VV modes with different mode orders. The uncertainties represent one SD derived from multiple repeated measurements.

Owing to the orthogonality of VV modes, when both the input probe and conjugate fields are in vacuum states, this amplifier can generate a large number of independent two-mode squeezed vacuum states, each containing a VV mode in the probe field and the corresponding VV mode with the same mode orders in the conjugate field, within the output twin beams. These individually addressable VV mode pairs can be exploited to produce multiple channels of independent two-mode CV entanglement. To verify the CV entanglement between VV modes, two sets of BHDs, one for the probe and the other for the conjugate, are used. Each BHD performs a projection measurement of the output multimode optical field onto a specific VV mode, determined by the local oscillator prepared according to Eq. 4 (see Materials and Methods for details about the experimental setup). This allows us to selectively project the probe and conjugate fields onto chosen VV modes and measure the corresponding quadratures to test CV entanglement. The experimentally obtained symplectic eigenvalues for different orders of VV modes are presented in Fig. 4. It can be observed that the symplectic eigenvalues increase with ∣ℓ∣, indicating a decreasing degree of CV entanglement. This reduction in degree of entanglement is attributed to the weaker interaction strength for higher ∣ℓ∣, as discussed earlier. Under our experimental conditions, as shown in Fig. 4, the symplectic eigenvalues for VV modes with ∣ℓ∣≤4 and both ± combinations are all below one, confirming the generation of 16 sets of entangled VV mode pairs from this amplifier. More specifically, each set of entanglement consists of two VV modes with the same mode orders (ℓ,±), one in the probe field and the other in the conjugate field.

## DISCUSSION

In summary, we propose a polarization-insensitive amplifier based on the FWM process in hot ^85^Rb atomic vapor, enabling the direct generation of massive VV modes exhibiting two-mode CV entanglement. By leveraging the independent conservation of both SAM and OAM in the FWM process, the amplifier is capable of amplifying VV optical modes with spatially inhomogeneous polarization. The output optical fields are further quantitatively characterized by OAM modal analysis under circular polarization projection and VQF measurements. When seeded by vacuum fields, the amplifier generates massive sets of independent two-mode squeezed vacuum states, each containing two VV modes with the same mode orders. Using the PPT criterion, we experimentally verify the generation of 16 pairs of entangled VV modes. The number of generated entangled VV mode pairs is limited by pump beam waist, nonlinear medium length, and experimental loss and noise. Increasing the pump beam waist or shortening the nonlinear medium length (*30*, *36*, *37*), as well as tailoring the pump beam profile to improve mode overlap, for example, using flat-top profiles (*38*), could potentially increase the number of accessible modes in future studies.

Although we focus on the CV quantum properties of VV modes in this work, it is important to clarify the distinction between CV (*31*) and discrete-variable (DV) (*39*) entanglement in the context of the present system. In the CV regime studied here, the FWM process operates in the strong interaction regime, and BHD is used to access quadrature correlations between pairs of VV modes. By contrast, DV entanglement relies on correlations between discrete-level states of individual photons and is typically measured via coincidence detection (*40*–*42*). In principle, by operating the polarization-insensitive amplifier in the weak interaction regime and using photon-counting measurements, the system could also be adapted to generate two-photon high-dimensional DV entanglement in the VV mode basis.

In addition, the polarization-insensitive amplifier provides a promising platform for studying more general vectorially structured modes beyond the cylindrically symmetric VV modes considered here. In particular, with appropriate engineering to achieve comparable intensity gain across different OAM orders, the amplifier can support superpositions of two arbitrary OAM modes with orthogonal circular polarizations, analogous to the output states of J-plates (*34*, *43*). Such generalized modes can constitute the building blocks for optical topological quasiparticles with complex polarization textures, including optical skyrmions (*44*–*46*). Therefore, the polarization-insensitive amplifier could, in principle, be extended to generate entanglement between such optical topological quasiparticles, for example, entangled optical skyrmions, opening opportunities to explore topological photonics and quantum technologies.

Moreover, since the FWM process based on this energy level configuration has already realized numerous amplifier-based quantum information protocols, such as quantum cloning (*47*) and quantum teleportation (*48*), the polarization-insensitive amplifier proposed here could facilitate quantum information protocols that fully harness the vectorial nature of optical fields. Our work establishes a paradigm for the direct generation of massive vector-mode entanglement, potentially advancing applications of vectorially structured modes in quantum optical technologies.

## MATERIALS AND METHODS

### Detailed experimental setup

**Fig. 5.**
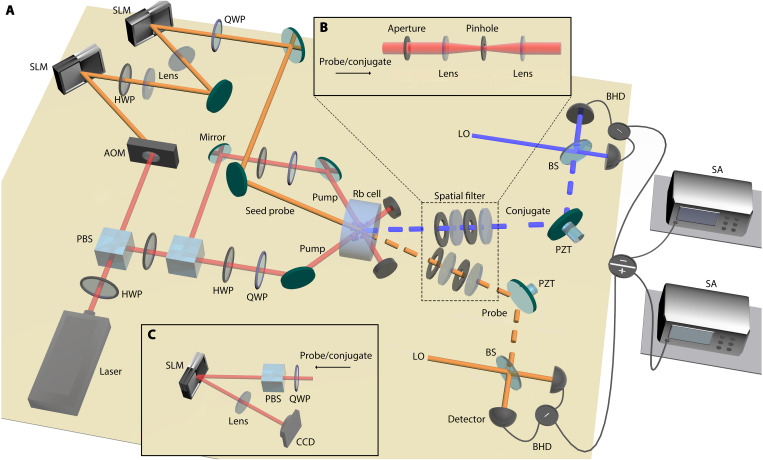
Detailed experimental setup. (**A**) Schematic of the generation and verification of entangled VV modes. (**B**) Details of the spatial filter using a 4-f imaging system consisting of two lenses, an aperture, and a pinhole, providing nonpolarizing pump filtering. (**C**) Setup for measuring VQF and OAM modal analysis. BS, beam splitter; HWP, half-wave plate; LO, local oscillator; PBS, polarizing beam splitter; PZT, piezoelectric transducer; SA, spectrum analyzer.

The detailed experimental setup is shown in Fig. 5. A Ti:sapphire laser generates a Gaussian beam whose frequency is stabilized at 377.1101 THz. This beam is divided, with one portion split further into two pump beams for the FWM process, as indicated by the red lines in Fig. 5A. Each pump beam has an optical power of 300 mW and a radius of ~415 μm. The other portion from the Ti:sapphire laser is red-shifted by 3.046 GHz via an acousto-optic modulator (AOM) to serve as the seed probe beam, shown by the yellow line in Fig. 5A. To convert the seed probe beam into VV modes (ℓ,±) as defined in Eq. 4, two spatial light modulators (SLMs), which respond only to the horizontal polarization component, are used. The horizontally polarized seed probe beam first passes through an SLM with a computer-generated hologram for the ${\text{LG}}_{\ell }$ mode, and the diffracted light then enters a 4-f imaging system with spatial filtering at the Fourier plane. The transmitted first-order diffracted light is transformed into diagonal polarization (equal superposition of horizontal and vertical polarization components) by a half-wave plate and enters a second SLM at the image plane. This second SLM applies a phase profile of exp(−2iℓϕ) or exp(−2iℓϕ+π) for generating VV modes (ℓ,+) and (ℓ,−), respectively. As a result, the horizontal and vertical polarization components of the beam share the same Laguerre-Gaussian amplitude profile but have opposite topological charges and a phase difference of 0 or π. The beam is then passed through a quarter-wave plate (QWP), converting the horizontal and vertical polarization components into left- and right-circular polarization components, respectively, thereby producing a seed probe beam with controllable VV mode orders.

The seed probe beam intersects symmetrically with the two pump beams at the center of an ^85^Rb vapor cell which is 12 mm long, generating the conjugate beam, represented by the blue line in Fig. 5A. To preserve polarization insensitivity, nonpolarizing pump filtering is implemented by exploiting the natural spatial separation between the interacting beams, followed by additional spatial filtering using 4-f imaging systems. The output probe and conjugate beams from the vapor cell propagate noncollinearly with respect to the two pump beams, so most of the pump light is blocked immediately after the vapor cell using beam blocks. For residual scattered pump light, each detection path for output probe and conjugate beams includes a 4-f imaging system composed of two lenses with an aperture placed at the image plane and a pinhole at the Fourier plane, which together act as a spatial filter (Fig. 5B). The aperture and pinhole diameters were optimized to maximize pump rejection while minimizing loss for the probe and conjugate beams as much as possible. Last, the output probe and conjugate beams are directed for polarization analysis, OAM modal analysis, VQF measurement, intensity gain measurement, and entanglement verification.

To generate massive two-mode squeezed vacuum states of VV modes, the seed probe beam is blocked before the ^85^Rb vapor cell, allowing the FWM process to operate in the vacuum-seeded regime. Two sets of BHDs are used to measure the amplitude and phase quadratures of the generated probe and conjugate fields. The local oscillators for the BHDs are created using another identical FWM process seeded by a bright probe beam as previously described, where the amplified probe and the generated conjugate beams from this seeded FWM process are used as local oscillators for the probe and conjugate BHDs, respectively. A 50:50 beam splitter combines the measured field with the local oscillator. A piezoelectric transducer changes the phase difference between the measured field and the local oscillator to be 0 or π/2, enabling the measurement of the amplitude and phase quadratures, respectively. The photocurrents from the BHDs are recorded by two spectrum analyzers set to 300-kHz resolution bandwidth, 300-Hz video bandwidth, zero span, and 1.5-MHz center frequency.

### Quantitative characterization of VV modes

The VQF provides a quantitative measure of the degree of vectorness of optical fields. Following the methodology developed in (*35*), the VQF is defined as

$$\text{VQF}=\text{Re}\left(\sqrt{1-s^{2}}\right)$$ (6)

where $s={\left(\sum_{i}{\langle \sigma_{i}\rangle }^{2}\right)}^{1/2}$ is the Bloch-vector length constructed from the expectation values of the Pauli operators $\left\langle \sigma_{i}\right\rangle$, obtained from normalized projection intensities. To evaluate the VQF, for each VV mode (ℓ,±), we measured 12 normalized intensities corresponding to six spatial-mode projections performed under two polarization basis states (left- and right-circular), yielding intensity values $I_{\mathrm{ij}}$. The six spatial-mode projections include two pure OAM modes, exp(iℓϕ) and exp(−iℓϕ), and four balanced superpositions, exp(iℓφ)+exp(iα)exp(−iℓφ), with relative phases α∈{0,π/2,π,3π/2}. The correspondence between projections and measured intensities is summarized in Table 1. From these normalized intensities, the expectation values of the Pauli operators are calculated as

$$\begin{array}{c} \langle \sigma_{1}\rangle =\left(I_{13}+I_{23}\right)-\left(I_{15}+I_{25}\right),\\ \langle \sigma_{2}\rangle =\left(I_{14}+I_{24}\right)-\left(I_{16}+I_{26}\right),\\ \langle \sigma_{3}\rangle =\left(I_{11}+I_{21}\right)-\left(I_{12}+I_{22}\right) \end{array}$$ (7)

from which s and hence the VQF are obtained. As illustrated in Fig. 5C, the polarization projection onto left- and right-circular polarizations is realized using a QWP followed by a polarizing beam splitter (PBS). Setting the QWP fast axis at 45° (−45°) converts right- (left-) circular polarization to horizontal linear polarization, which is transmitted through the PBS. The transmitted field is then analyzed by the spatial-mode projection consisting of a SLM displaying the appropriate hologram, a lens, and a CCD camera. The on-axis intensity in the Fourier plane is recorded and corresponds to the projection onto the selected spatial mode.

**Table 1.** VQF measurements. Normalized projection intensities $I_{\mathrm{ij}}$ used to evaluate the VQF of a VV mode (ℓ,±).

Basis state	exp(iℓϕ)	exp(−iℓϕ)	α=0	α=π/2	α=π	α=3π/2
L	$I_{11}$	$I_{12}$	$I_{13}$	$I_{14}$	$I_{15}$	$I_{16}$
R	$I_{21}$	$I_{22}$	$I_{23}$	$I_{24}$	$I_{25}$	$I_{26}$

The OAM modal analysis of VV modes under right- and left-circular polarization projections is implemented using the same detection path as in the VQF measurement (Fig. 5C). After selecting the desired polarization projection, the OAM mode projections were sequentially varied, and the corresponding normalized on-axis Fourier plane intensities were recorded to obtain the OAM spectrum.

## Supplementary Materials

### This PDF file includes:

Sections S1 to S4 Figs. S1 to S6
