# Supplementary material for: Direct generation of massive vector-mode entanglement from a polarization-insensitive optical amplifier
Source: Sci Adv. 2026 Apr 17;12(16):eaec0001. doi: 10.1126/sciadv.aec0001 (PMC13089346; doi:10.1126/sciadv.aec0001)
Supplement: Supplementary file 1 — Sections S1 to S4 Figs. S1 to S6 [file sciadv.aec0001_sm.pdf]

Science Advances
AAAS

Supplementary Materials for

Direct generation of massive vector-mode entanglement from a polarization-insensitive optical amplifier

Xutong Wang *et al.*

Corresponding author: Jietai Jing, jtjing@phy.ecnu.edu.cn

Sci. Adv. **12**, eaec0001 (2026)
DOI: 10.1126/sciadv.aec0001

This PDF file includes:

Sections S1 to S4
Figs. S1 to S6

Supplementary Text

S1. Effect of pump polarizations on polarization sensitivity

In our setup, the two pump beams carry orthogonal circular polarizations, enabling the realization of a polarization-insensitive amplifier. By contrast, when both pump beams (P_1 and P_2) have the same circular polarization, spin angular momentum (SAM) conservation in the four-wave mixing (FWM) process, expressed as $\sigma_{\mathrm{P}_1} + \sigma_{\mathrm{P}_2} = \sigma_{\mathrm{Pr}} + \sigma_{\mathrm{C}}$ (with $\sigma = \pm 1$ for left-/right-circular polarization), requires that the probe (Pr) and conjugate (C) beams carry the same circular polarization as the pump beams. If the probe beam carries the opposite circular polarization to pump beams, SAM conservation cannot be satisfied for any conjugate polarization, and the FWM process is therefore forbidden. In this case the interaction obeys a definite polarization selection rule and is no longer polarization-insensitive, so entangled vector vortex (VV) modes cannot be generated.

To verify this polarization selection rule experimentally, we perform measurements, in which both pump beams are right-circularly polarized and a bright beam with varying polarizations is seeded into the amplifier along the direction of the probe beam, and analyze the polarizations of the output probe and conjugate beams. The intensity of different polarization components, normalized to the intensity of the seed probe beam, for the output probe and conjugate beams is shown in Fig. S1A. When the seed probe is right-circularly polarized, the amplified probe and the newly generated conjugate beams both remain right-circularly polarized. When the seed probe is left-circularly polarized, the FWM process is forbidden: the seed probe is not amplified, and no conjugate beam appears. A complementary test with both pump beams left-circularly polarized shows the same behavior: FWM process occurs only when the seed probe is left-circularly polarized (Fig. S1B). These experimental results confirm the predicted polarization selectivity of the case with two pump beams having the same circular polarization. Therefore, we utilize two pump beams with orthogonal circular polarizations to construct the polarization-insensitive amplifier.

S2. Suppression of single-pump FWM processes

In our configuration, other potential FWM processes, particularly the single-pump FWM (*29*), are strongly suppressed by the chosen beam geometry. The two pump beams intersect at 17.4 mrad, while the probe and conjugate beams propagate at 8.5 mrad from the pump-beam plane. Consequently, for the single-pump FWM processes, the angle between pump beam and the probe/conjugate beams is about 12.2 mrad, well above the typical phase-matching angle (≈ 2-10 mrad) (*29, 33*). Such a mismatch makes single-pump FWM processes negligible.

We verified this experimentally by seeding a bright beam along the direction of the probe beam (Pr) into the Rb atomic vapor cell and recording the output intensity distributions (Fig. S2). The conjugate beams C_1 and C_2 that would result from single-pump FWM processes (P_1 or P_2 alone) are negligible and several orders of magnitude weaker than the conjugate C generated by the two-pump polarization-insensitive configuration. Similarly, the probe beams Pr_1 and Pr_2 from single-pump FWM processes are also negligible. These results confirm that single-pump FWM processes are effectively suppressed in our system.

S3. Theoretical derivation of input-output relations and entanglement for paired VV modes

As in the main text, the Hamiltonian describing our system in the interaction picture can be expressed as

$$\hat{H} = \sum_{\ell} i\hbar\gamma_\ell \left(\hat{a}^\dagger_{R,\ell}\hat{b}^\dagger_{L,-\ell} + \hat{a}^\dagger_{L,\ell}\hat{b}^\dagger_{R,-\ell}\right) + \mathrm{H.C.}, \tag{S1}$$

where $\hat{a}^\dagger_{R,\ell}$ ($\hat{a}^\dagger_{L,\ell}$) and $\hat{b}^\dagger_{L,-\ell}$ ($\hat{b}^\dagger_{R,-\ell}$) are the creation operators for probe and conjugate modes, respectively, with the two subscripts denoting their SAM (R, right-circular polarization; L, left-circular polarization) and OAM (ℓ, topological charge), γ_ℓ represents the corresponding interaction strength, and $\mathrm{H.C.}$ is the Hermitian conjugate. The corresponding input-output relations in such SAM-OAM basis for the probe and conjugate fields can be written as

$$\begin{aligned} \hat{a}^\dagger_{R,\ell} &= \sqrt{G_\ell}\hat{c}^\dagger_{R,\ell} + \sqrt{G_\ell - 1}\hat{d}_{L,-\ell}, \\ \hat{b}^\dagger_{L,-\ell} &= \sqrt{G_\ell - 1}\hat{c}_{R,\ell} + \sqrt{G_\ell}\hat{d}^\dagger_{L,-\ell}, \end{aligned} \tag{S2}$$

and

$$\begin{aligned} \hat{a}^\dagger_{L,\ell} &= \sqrt{G_\ell}\hat{c}^\dagger_{L,\ell} + \sqrt{G_\ell - 1}\hat{d}_{R,-\ell}, \\ \hat{b}^\dagger_{R,-\ell} &= \sqrt{G_\ell - 1}\hat{c}_{L,\ell} + \sqrt{G_\ell}\hat{d}^\dagger_{R,-\ell}, \end{aligned} \tag{S3}$$

where $\hat{c}^\dagger_{R,\ell}$ ($\hat{c}^\dagger_{L,\ell}$) and $\hat{d}^\dagger_{L,-\ell}$ ($\hat{d}^\dagger_{R,-\ell}$) represent the creation operators for the input probe and conjugate modes, respectively, and $G_\ell = \cosh^2(\gamma_\ell \tau)$ is the intensity gain with interaction time τ.

We consider a class of VV modes expressed in the Laguerre–Gaussian (LG) mode basis as

$$\mathbf{E}_{\ell,\pm}(r,\phi,z) = \mathrm{LG}_\ell(r,\phi,z)\boldsymbol{e}_R \pm \mathrm{LG}_{-\ell}(r,\phi,z)\boldsymbol{e}_L, \tag{S4}$$

where $\boldsymbol{e}_R$ and $\boldsymbol{e}_L$ represent the basis vectors of the right- and left-circular polarizations, and $\mathrm{LG}_\ell(r,\phi,z)$ denotes a LG mode with topological charge ℓ and a radial index of 0. According to this VV mode structure, we now need to reverse the sign of the topological charge ℓ in Eq. (S3), which gives

$$\begin{aligned} \hat{a}^\dagger_{L,-\ell} &= \sqrt{G_{-\ell}}\hat{c}^\dagger_{L,-\ell} + \sqrt{G_{-\ell} - 1}\hat{d}_{R,\ell}, \\ \hat{b}^\dagger_{R,\ell} &= \sqrt{G_{-\ell} - 1}\hat{c}_{L,-\ell} + \sqrt{G_{-\ell}}\hat{d}^\dagger_{R,\ell}, \end{aligned} \tag{S5}$$

and add/subtract the input-output relations in the SAM-OAM basis [Eqs. (S2) and (S5)] from both sides to obtain input-output relations for VV modes. Assuming equal intensity gain for opposite OAM orders ($G_\ell = G_{-\ell}$), we obtain

$$\begin{aligned} \hat{a}^\dagger_{R,\ell} \pm \hat{a}^\dagger_{L,-\ell} &= \sqrt{G_\ell}\left(\hat{c}^\dagger_{R,\ell} \pm \hat{c}^\dagger_{L,-\ell}\right) + \sqrt{G_\ell - 1}\left(\hat{d}_{L,-\ell} \pm \hat{d}_{R,\ell}\right), \\ \hat{b}^\dagger_{L,-\ell} \pm \hat{b}^\dagger_{R,\ell} &= \sqrt{G_\ell - 1}\left(\hat{c}_{R,\ell} \pm \hat{c}_{L,-\ell}\right) + \sqrt{G_\ell}\left(\hat{d}^\dagger_{L,-\ell} \pm \hat{d}^\dagger_{R,\ell}\right). \end{aligned} \tag{S6}$$

According to Eq. (S4), the creation operators for VV modes ($\hat{f}^\dagger_{\ell,\pm}$, $\hat{g}^\dagger_{\ell,\pm}$, $\hat{h}^\dagger_{\ell,\pm}$, and $\hat{\imath}^\dagger_{\ell,\pm}$) follow a unitary transformation of the above SAM-OAM basis:

$$\begin{aligned} \hat{f}^\dagger_{\ell,\pm} &= \frac{\hat{a}^\dagger_{R,\ell} \pm \hat{a}^\dagger_{L,-\ell}}{\sqrt{2}}, \\ \hat{g}^\dagger_{\ell,\pm} &= \frac{\hat{b}^\dagger_{L,-\ell} \pm \hat{b}^\dagger_{R,\ell}}{\sqrt{2}}, \end{aligned} \tag{S7}$$

for the output VV modes in the probe and conjugate fields, and

$$\hat{h}^{\dagger}_{\ell,\pm} = \frac{\hat{c}^{\dagger}_{R,\ell} \pm \hat{c}^{\dagger}_{L,-\ell}}{\sqrt{2}},$$
$$\hat{\iota}^{\dagger}_{\ell,\pm} = \frac{\hat{d}^{\dagger}_{L,-\ell} \pm \hat{d}^{\dagger}_{R,\ell}}{\sqrt{2}}, \tag{S8}$$

for the corresponding input VV modes. Substituting these definitions into Eq. (S6), we obtain the input-output relations in the VV mode basis:

$$\hat{f}^{\dagger}_{\ell,\pm} = \sqrt{G_\ell}\hat{h}^{\dagger}_{\ell,\pm} + \sqrt{G_\ell - 1}\hat{\iota}_{\ell,\pm},$$
$$\hat{g}^{\dagger}_{\ell,\pm} = \sqrt{G_\ell - 1}\hat{h}_{\ell,\pm} + \sqrt{G_\ell}\hat{\iota}^{\dagger}_{\ell,\pm}. \tag{S9}$$

This derivation explicitly shows how the general description [Eqs. (S1-S3)] lead to corresponding input-output relations for paired VV modes [Eqs. (S9)], through a unitary basis change according to the expression of VV modes [Eq. (S4)]. The polarization-insensitive amplifier thus mixes and amplifies VV mode pairs sharing the same mode orders $(\ell, \pm)$.

For generating CV quantum entanglement, the input probe and conjugate fields are in vacuum. The amplifier then generates quantum-correlated twin fields (probe and conjugate), each containing massive VV modes. We study CV entanglement between two VV modes with the same $(\ell, \pm)$, one in the probe and one in the conjugate. Each VV mode is described quantum-mechanically by a pair of quadrature operators, amplitude quadrature

$$\hat{x}_{Pr,\ell,\pm} = \hat{f}_{\ell,\pm} + \hat{f}^{\dagger}_{\ell,\pm},$$
$$\hat{x}_{C,\ell,\pm} = \hat{g}_{\ell,\pm} + \hat{g}^{\dagger}_{\ell,\pm}, \tag{S10}$$

and phase quadrature

$$\hat{p}_{Pr,\ell,\pm} = i(\hat{f}^{\dagger}_{\ell,\pm} - \hat{f}_{\ell,\pm}),$$
$$\hat{p}_{C,\ell,\pm} = i(\hat{g}^{\dagger}_{\ell,\pm} - \hat{g}_{\ell,\pm}). \tag{S11}$$

Each pair of VV modes ($\hat{f}_{\ell,\pm}$ and $\hat{g}_{\ell,\pm}$) forms a two-mode squeezed vacuum state, i.e. a CV entangled state. For such a state, there are correlations between the amplitude quadratures and anti-correlations between the phase quadratures of the two VV modes. Owing to the orthogonality of the VV modes, these CV entangled states for different $(\ell, \pm)$ are generated simultaneously and in parallel, enabling the generation of multiple sets of independent CV entanglement between paired VV modes.

The CV entanglement of a given VV mode pair can be analyzed using the covariance matrix (CM) constructed from the quadratures

$$V_{\ell,\pm} = \begin{bmatrix} \langle \hat{x}_{Pr,\ell,\pm}\,\hat{x}_{Pr,\ell,\pm}\rangle & \langle \hat{x}_{Pr,\ell,\pm}\,\hat{p}_{Pr,\ell,\pm}\rangle & \langle \hat{x}_{Pr,\ell,\pm}\,\hat{x}_{C,\ell,\pm}\rangle & \langle \hat{x}_{Pr,\ell,\pm}\,\hat{p}_{C,\ell,\pm}\rangle \\ \langle \hat{p}_{Pr,\ell,\pm}\,\hat{x}_{Pr,\ell,\pm}\rangle & \langle \hat{p}_{Pr,\ell,\pm}\,\hat{p}_{Pr,\ell,\pm}\rangle & \langle \hat{p}_{Pr,\ell,\pm}\,\hat{x}_{C,\ell,\pm}\rangle & \langle \hat{p}_{Pr,\ell,\pm}\,\hat{p}_{C,\ell,\pm}\rangle \\ \langle \hat{x}_{C,\ell,\pm}\,\hat{x}_{Pr,\ell,\pm}\rangle & \langle \hat{x}_{C,\ell,\pm}\,\hat{p}_{Pr,\ell,\pm}\rangle & \langle \hat{x}_{C,\ell,\pm}\,\hat{x}_{C,\ell,\pm}\rangle & \langle \hat{x}_{C,\ell,\pm}\,\hat{p}_{C,\ell,\pm}\rangle \\ \langle \hat{p}_{C,\ell,\pm}\,\hat{x}_{Pr,\ell,\pm}\rangle & \langle \hat{p}_{C,\ell,\pm}\,\hat{p}_{Pr,\ell,\pm}\rangle & \langle \hat{p}_{C,\ell,\pm}\,\hat{x}_{C,\ell,\pm}\rangle & \langle \hat{p}_{C,\ell,\pm}\,\hat{p}_{C,\ell,\pm}\rangle \end{bmatrix}$$
$$= \begin{bmatrix} 2G_\ell - 1 & 0 & 2\sqrt{G_\ell(G_\ell - 1)} & 0 \\ 0 & 2G_\ell - 1 & 0 & -2\sqrt{G_\ell(G_\ell - 1)} \\ 2\sqrt{G_\ell(G_\ell - 1)} & 0 & 2G_\ell - 1 & 0 \\ 0 & -2\sqrt{G_\ell(G_\ell - 1)} & 0 & 2G_\ell - 1 \end{bmatrix}. \tag{S12}$$

From this CM, the smallest symplectic eigenvalue of the partially transposed CM is

$$\nu_{\ell,\pm} = \sqrt{1 + 8G_\ell(G_l - 1) - 4\sqrt{G_\ell(G_\ell - 1)(2G_\ell - 1)^2}}. \tag{S13}$$

According to the positivity under partial transposition (PPT) criterion (*32*), $\nu_{\ell,\pm} < 1$ certifies CV entanglement, and a smaller value of $\nu_{\ell,\pm}$ corresponds to a stronger degree of CV entanglement. It can be found that $\nu_{\ell,\pm} < 1$ for any $G_\ell > 1$, confirming the generation of CV entanglement between paired VV modes.

S4. Experimental results for the quantitative characterization of VV modes

Figs. S3 and S4 provide the OAM modal analysis results for all the VV modes studied in the main text. Figs. S5 and S6 provide the measured normalized projection intensities used to evaluate the vector quality factor values, which are summarized in Fig. 3D of the main text, for all the VV modes studied in the main text.

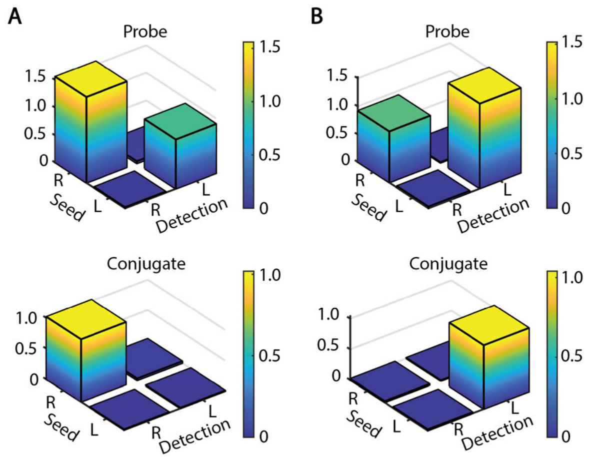

Fig. S1.

Polarization analysis of the output probe (upper panel) and conjugate (lower panel) beams from the amplifier when both pump beams are (**A**) right- and (**B**) left-circularly polarized.

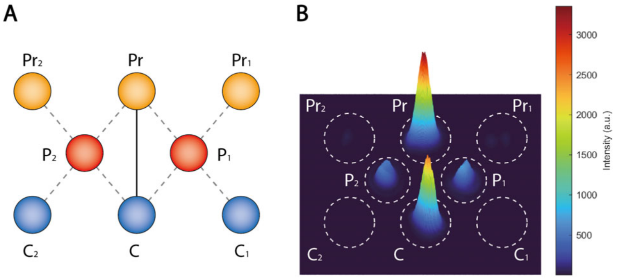

Fig. S2.

Suppression of single-pump FWM processes. (**A**) Schematic of two-pump (solid line) and potential single-pump (dashed lines) FWM configurations. The large pump-probe angles exceed the phase-matching angle, preventing single-pump FWM processes. (**B**) Experimental intensity distributions of the output fields after seeding a bright probe beam, showing negligible conjugate beam generation from single-pump FWM processes. The two pump beams are strongly attenuated after the vapor cell to prevent overexposure of the CCD camera.

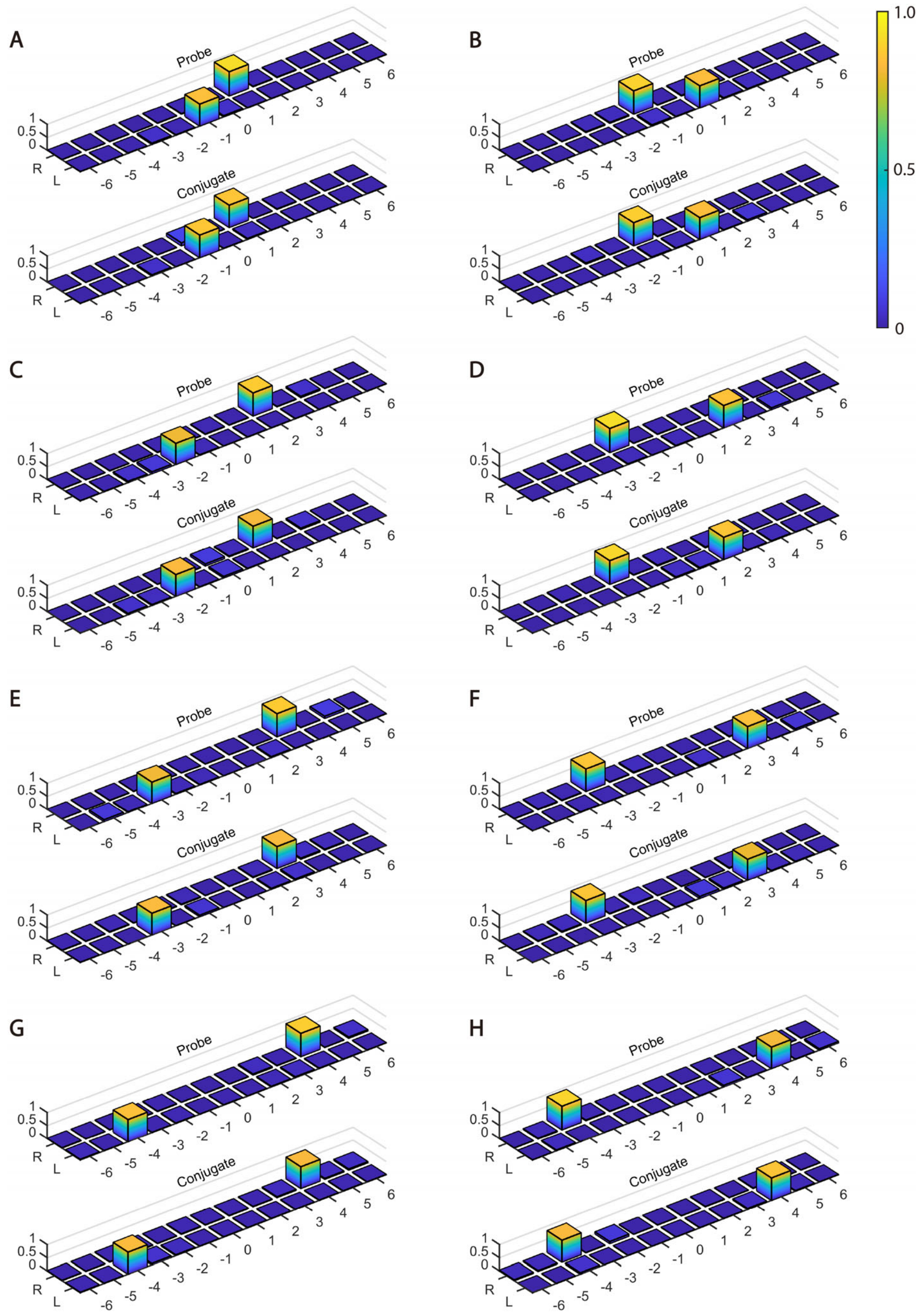

Fig. S3.

OAM modal analysis of the generated VV modes $(\ell, +)$ under circular polarization projection. (**A**) $\ell = 1$; (**B**) $\ell = -1$; (**C**) $\ell = 2$; (**D**) $\ell = -2$; (**E**) $\ell = 3$; (**F**) $\ell = -3$; (**G**) $\ell = 4$; (**H**) $\ell = -4$.

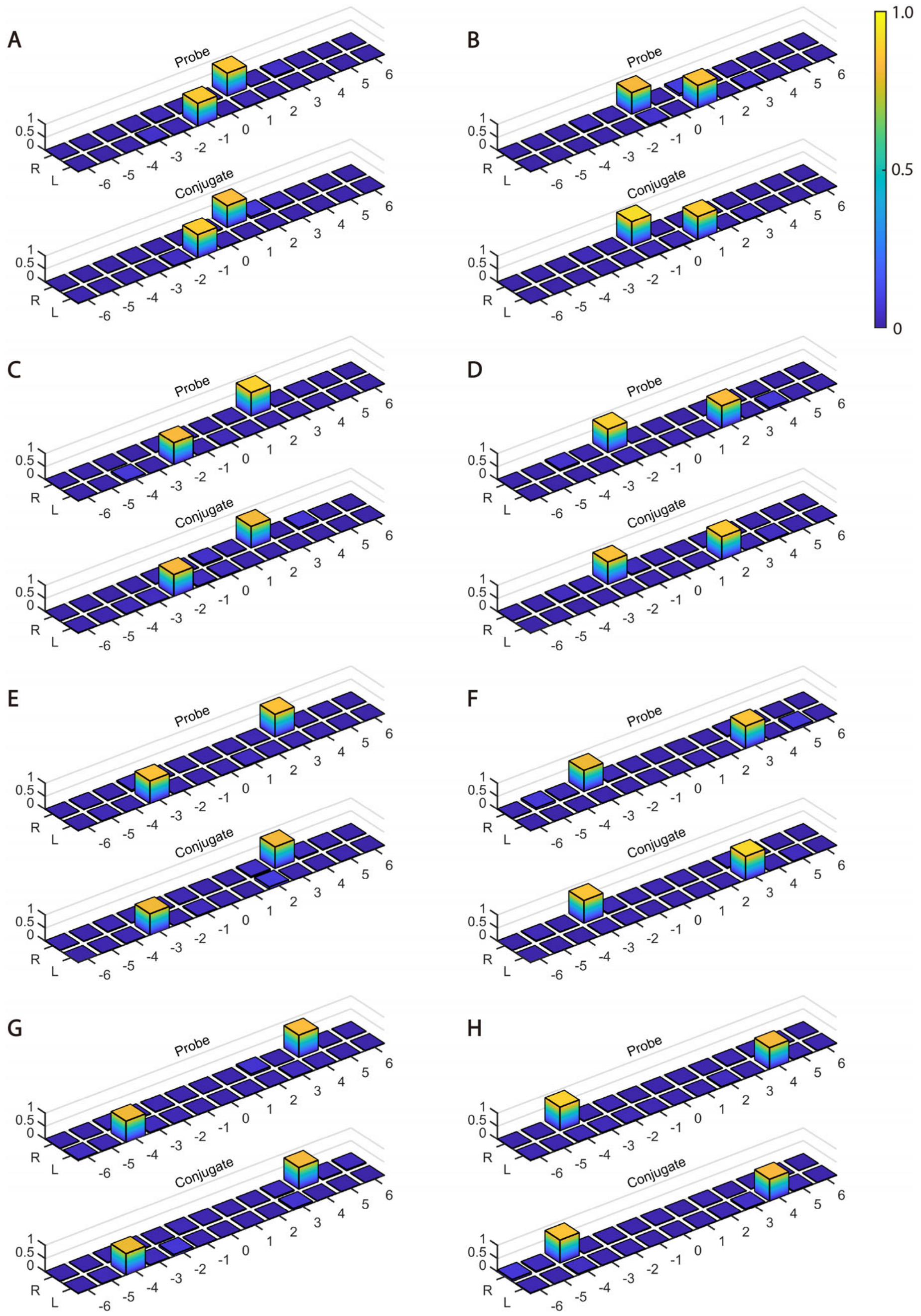

Fig. S4.

OAM modal analysis of the generated VV modes $(\ell, -)$ under circular polarization projection. (**A**) $\ell = 1$; (**B**) $\ell = -1$; (**C**) $\ell = 2$; (**D**) $\ell = -2$; (**E**) $\ell = 3$; (**F**) $\ell = -3$; (**G**) $\ell = 4$; (**H**) $\ell = -4$.

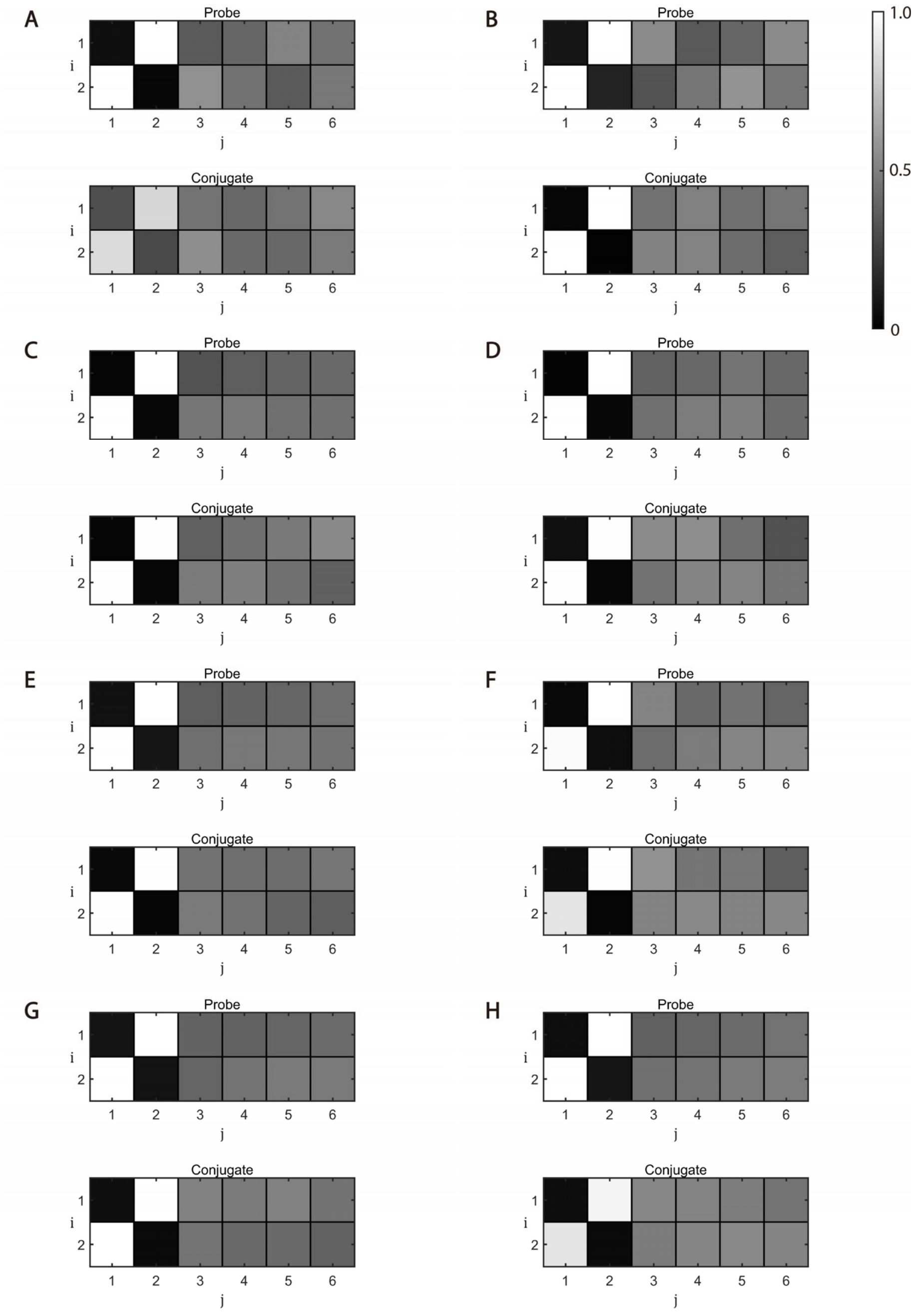

Fig. S5.

Measured normalized projection intensities I_{ij} used to evaluate the vector quality factor for the generated VV modes $(\ell, +)$. The indices i and j follow the definitions given in Table 1 of the main text. (**A**) $\ell = 1$; (**B**) $\ell = -1$; (**C**) $\ell = 2$; (**D**) $\ell = -2$; (**E**) $\ell = 3$; (**F**) $\ell = -3$; (**G**) $\ell = 4$; (**H**) $\ell = -4$.

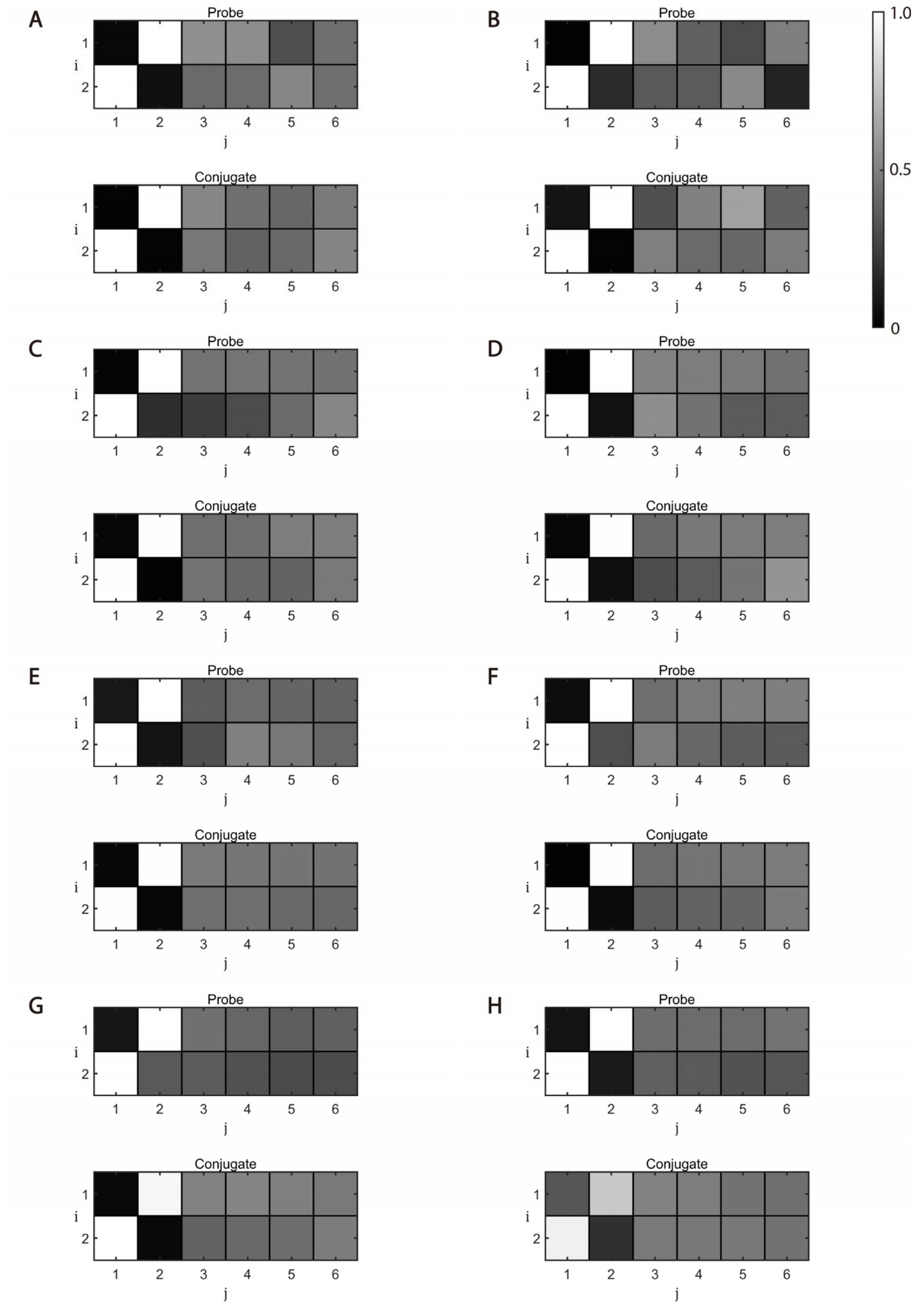

Fig. S6.

Measured normalized projection intensities I_{ij} used to evaluate the vector quality factor for the generated VV modes $(\ell, -)$. The indices i and j follow the definitions given in Table 1 of the main text. (**A**) $\ell = 1$; (**B**) $\ell = -1$; (**C**) $\ell = 2$; (**D**) $\ell = -2$; (**E**) $\ell = 3$; (**F**) $\ell = -3$; (**G**) $\ell = 4$; (**H**) $\ell = -4$.
